# A Review of Wearable Optical Fiber Sensors for Rehabilitation Monitoring

**DOI:** 10.3390/s24113602

**Published:** 2024-06-03

**Authors:** Xiangmeng Li, Yongzhen Li, Huifen Wei, Chaohui Wang, Bo Liu

**Affiliations:** Shanxi Provincial Key Laboratory for Advanced Manufacturing Technology, North University of China, Taiyuan 030051, China; s202302017@st.nuc.edu.cn (Y.L.); huifenwei@nuc.edu.cn (H.W.); s202202014@st.nuc.edu.cn (C.W.); s202302019@st.nuc.edu.cn (B.L.)

**Keywords:** wearable optical fiber sensor, rehabilitation, monitoring

## Abstract

As the global aging population increases, the demand for rehabilitation of elderly hand conditions has attracted increased attention in the field of wearable sensors. Owing to their distinctive anti-electromagnetic interference properties, high sensitivity, and excellent biocompatibility, optical fiber sensors exhibit substantial potential for applications in monitoring finger movements, physiological parameters, and tactile responses during rehabilitation. This review provides a brief introduction to the principles and technologies of various fiber sensors, including the Fiber Bragg Grating sensor, self-luminescent stretchable optical fiber sensor, and optic fiber Fabry–Perot sensor. In addition, specific applications are discussed within the rehabilitation field. Furthermore, challenges inherent to current optical fiber sensing technology, such as enhancing the sensitivity and flexibility of the sensors, reducing their cost, and refining system integration, are also addressed. Due to technological developments and greater efforts by researchers, it is likely that wearable optical fiber sensors will become commercially available and extensively utilized for rehabilitation.

## 1. Introduction

Based on the medium-variant projections from the United Nations’ “World Population Prospects 2022”, the share of the global population aged 65 and above is projected to rise from 10% to 16% by 2050. This suggests a global trend of population aging. By 2033, China, one of the most populous nations in the world, is expected to have over 400 million residents aged 60 and above, peaking at around 520 million by 2054. The trend of global population aging is evident both in the rising number of elderly individuals and their increasing share of the total population. For instance, countries like Italy, Finland, and Portugal have elderly populations exceeding 20% of their total populations [1]. As the elderly population ages, the risk of common diseases and accidents among this group is likely to rise significantly. For individuals with chronic hand conditions or disabilities, daily rehabilitation practices, including massage, acupuncture, and exercise therapy, are essential. Traditional rehabilitation treatments, confined to clinical settings, pose significant challenges for individuals with limited mobility. Currently, ongoing advancements in fields such as materials science, sensing technology, and communication engineering have led to the widespread adoption of wearable rehabilitation devices among patients with mobility impairments [2,3,4,5]. These devices offer patients flexible and continuous dynamic monitoring, supplying rehabilitation physicians with real-time data, thereby facilitating more convenient, remote treatment options during the rehabilitation process.

When the brain issues commands to move the fingers, it produces a range of biophysical signals, such as force, temperature, movement, and bioelectricity, as well as biochemical signals including hormones, lactic acid, cytokines, and changes in ion concentrations. Wearable sensors are capable of measuring and quantifying these signals, reflecting a range of hand physiological data, and aiding in the operation of wearable rehabilitation devices for hand treatment [6,7,8]. In the current market of wearable rehabilitation equipment, electronic sensors are predominantly utilized for detecting human physiological signals. Devices such as encoders, potentiometers, electrical goniometers, and linear variable differential transformers (LVDTs) are examples of electronic sensors that measure joint angular positions and segmental linear displacement [9]. However, the complexity of encoders necessitates meticulous manufacturing and precise installation. Additionally, they are susceptible to environmental factors like temperature, vibration, and dust, which can significantly increase measurement errors, thereby compromising the stability and reliability of wearable devices [10]. Potentiometers have a delicate mechanical structure that is vulnerable to both physical impacts and environmental humidity. Changes in humidity can degrade measurement precision and hasten the wear of the potentiometer materials, reducing its lifespan. Due to these sensitivities, potentiometers are ill-suited for wearable devices frequently experiencing motion or substantial stress [11]. Certain types of goniometers require frequent calibration to ensure data accuracy, which can increase the barriers to use and increase maintenance costs. Additionally, electric goniometers are susceptible to vibrations, which can cause output signal fluctuations and introduce uncertainty in the detection of human physiological signals for wearable devices requiring precise angular measurements [12]. LVDTs can suffer from issues like drift, reduced sensitivity, and limited resolution with prolonged use, necessitating regular calibration and maintenance. This requirement can escalate operational costs and system complexity, potentially rendering them unsuitable for applications demanding high precision and sensitivity [13]. Additionally, in the realm of wearable rehabilitation devices, resistive, capacitive, and piezoelectric sensors are predominantly utilized to measure physiological parameters, recognize gestures, monitor motion, and offer functionalities for data acquisition and user interaction. However, resistive, capacitive, and piezoelectric sensors are susceptible to damage. In particular, their sensitivity is highly affected by external environmental factors such as temperature and humidity fluctuations. An increase in temperature may cause thermal expansion of the sensor materials, affecting their resistance or capacitance values, and thereby reducing measurement accuracy. An increase in humidity may cause moisture condensation on the sensor surface or internally, which can not only lead to electrical short circuits but can also alter the electrical properties of the sensors, affecting the stability and reliability of the signal, and subsequently lead to issues such as increased system complexity, power consumption, and maintenance costs [9]. It is worth noting that when the actuator is operating the electromagnetic field it generates will simultaneously affect the real-time operation of the capacitive sensor, causing certain interference in the auxiliary control of wearable rehabilitation treatment devices for rehabilitation exercises and real-time monitoring of patient data [14]. Recent advancements in optical fiber sensing technology have established optical fiber sensors as viable alternatives to electronic sensors in wearable rehabilitation devices, emphasizing their key benefit: inherent immunity to electromagnetic interference. Furthermore, optical fiber sensors have found broad applications across the medical field [15,16,17], and optical fiber sensors are also utilized in sectors such as oil and gas [18], structural monitoring [19], environmental monitoring [20], aerospace [21,22], and industrial automation [23], benefited by their lightweight, compact size, high sensitivity, capability for remote measurement, ability to conduct multi-parameter measurements, and the capacity to integrate into sensor networks alongside other types of sensors.

**Figure 1:**
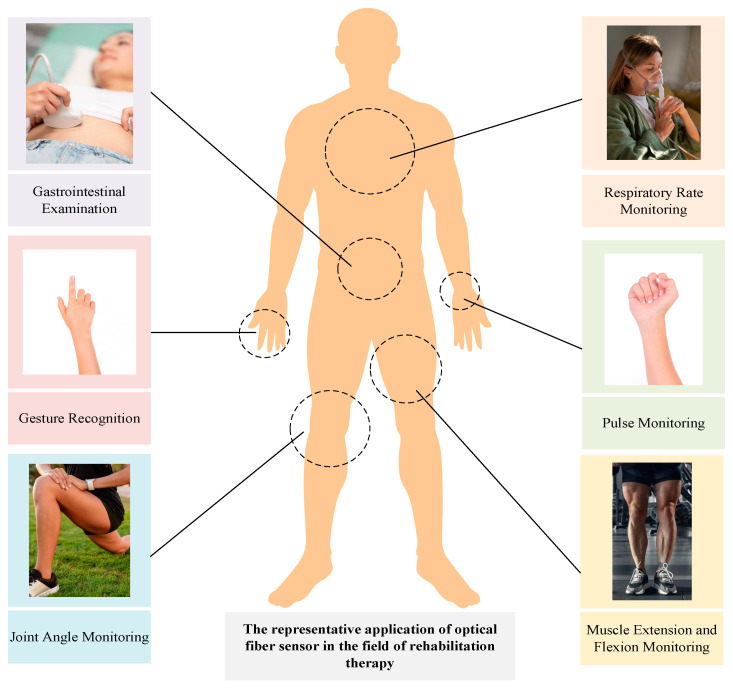
Representative applications for rehabilitation using wearable optical fiber sensors.

To meet the specific application requirements of wearable rehabilitation equipment, optical fiber sensors can be integrated with a variety of materials to create devices that are compatible with the human body and exhibit high sensitivity [24,25,26]. This includes specialized sensors such as optical fiber temperature sensors, optical fiber angle sensors, and optical fiber strain sensors. These sensors can be embedded into wearable items such as gloves, wristbands, chest straps, and medical shirts to monitor patients’ vital signs— including body temperature [27], pulse [28], respiratory rate [29], and exercise state [30,31]—in real time during rehabilitation. The data collected can then inform adjustments to the wearable rehabilitation equipment for optimal joint angles [32] and exercise modalities [33]. Representative applications of optical fiber sensors in the field of rehabilitation therapy are depicted in Figure 1. However, the widespread production and application of wearable optical fiber sensors in rehabilitation are currently limited due to their complex manufacturing processes and higher costs.

The detection of human finger parameters is crucial for both the recovery of muscle function and the assessment of hand motor skills during rehabilitation treatment. Wearable optical fiber sensors, offering a non-invasive, flexible approach with potential clinical applications, present a novel solution for rehabilitating hand function. However, applications in hand rehabilitation currently lack systematic analysis and the integration of key technologies. Thus, this paper reviews the technological advancements and potential applications within this domain. This paper discusses and summarizes the latest advancements in technology and finger parameter detection, drawing on the principles, fabrication methods, and materials of wearable optical fiber sensors. Section 2 primarily outlines the principles and fabrication methods of wearable optical fiber sensors. Section 3 presents case studies on the application of wearable optical fiber sensors for measuring human finger parameters. Section 4 discusses the challenges and prospective research directions for the implementation of wearable optical fiber sensors within the realm of finger parameter measurement. Section 5 offers a comprehensive summary of the entire review.

## 2. Working Principle of Wearable Optical Fiber Sensor

During the measurement of object parameters, wearable optical fiber sensors are subject to external influences, such as temperature and pressure. These influences can cause physical deformations like bending and stretching, which, in turn, alter the light transmission properties within the fibers. The sensor then detects these changes in the optical signal, which, upon processing using a photodetector and demodulator, are translated into the necessary measurement parameters. Presently, wearable optical fiber sensors primarily utilize fundamental physical properties like light intensity, wavelength, frequency, phase, and polarization for analyzing the characteristics of the objects under measurement. The diversity of optical fiber sensors is driving advancements in wearable rehabilitation equipment, offering patients a more user-friendly experience.

### 2.1. Fiber Bragg Grating Sensor

The Fiber Bragg Grating (FBG) sensor utilizes an internal grating etched into the optical fiber to convert optical signals into measurements indicative of stress, strain, or temperature. To mitigate the impact of environmental factors on the monitoring parameters, FBG sensors are typically encapsulated. Common encapsulation materials for FBG sensors include polymers [34,35], metals [36], and ceramics [37,38]. The choice of suitable encapsulation material is contingent upon the specific application environment and the performance demands of the sensor.

**Figure 2:**
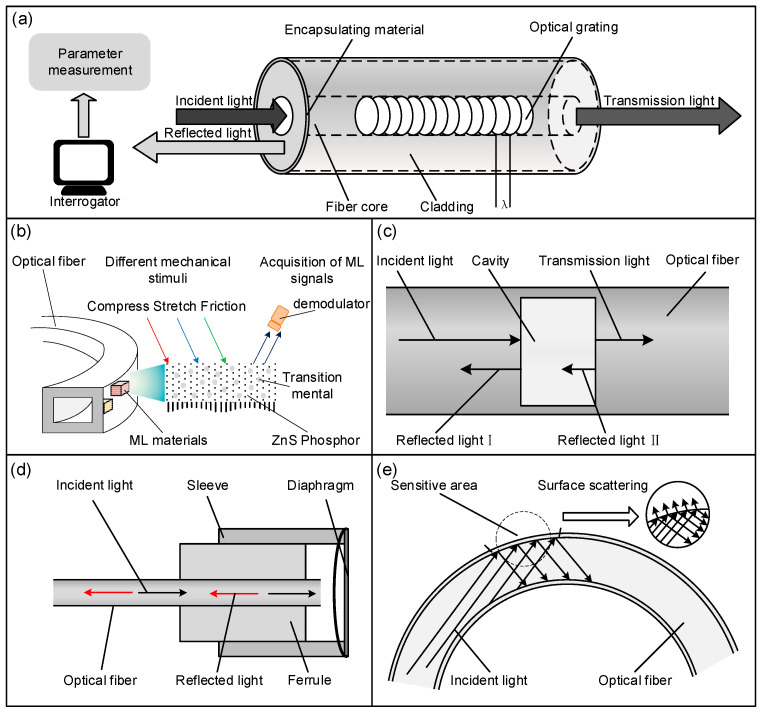
Schematic illustration of working principle for typical optical fiber sensors: (**a**) Fiber Bragg Grating sensor; (**b**) self-luminescent stretchable optical fiber sensor with mechanoluminescence (ML) materials; (**c**) optical fiber Fabry–Perot sensor; (**d**) diaphragm-type optical fiber sensor; and (**e**) polymer optical fiber sensor.

The working principle of the FBGs is illustrated in Figure 2a. When light propagates through an optical fiber, light at a specific central wavelength (the Bragg reflected light) is reflected at the Bragg grating region, while the rest of the wavelengths remain unaffected and continue to transmit. The reflected wavelength can be expressed as:

(1) $$\lambda =2\times n_{eff}\times \Lambda .$$

Among these parameters, λ represents the reflection wavelength of the FBGs, $n_{eff}$ is the effective refractive index of the grating, and Λ denotes the period of the grating fringe. Strain ε and temperature T can influence the central wavelength of the FBGs. The relationship between strain ε, temperature T, and the wavelength shift of the FBGs is described by the subsequent equation:

(2) $$\Delta \lambda =[(1-P_{e})\varepsilon +(\alpha +\xi )\Delta T]\lambda .$$

The changes encompass the central wavelength shift Δλ, strain ε, and temperature change ΔT, along with the photoelastic constant $P_{e}$, the thermal expansion coefficient α, and the thermo-optic coefficient ξ. FBGs offer distinct advantages for applications in structural, medical, and environmental monitoring, as well as in industrial process control, due to their adaptability to various environments and high measurement precision. They are especially well-suited for engineering applications that necessitate long-term stable monitoring. This establishes FBGs as a critical component of contemporary intelligent monitoring systems.

### 2.2. Self-Luminescent, Stretchable Optical Fiber Sensor

Currently, self-luminescent stretchable optical fiber sensors are typically fabricated using polymer casting and demolding techniques. Mechanoluminescence (ML) materials are strategically positioned within the fibers, with a demodulator attached at their extremities. Upon mechanical stimulation, the inherent self-luminescence of these materials is harnessed to produce internal sensor illumination, as depicted in Figure 2b.

Consider transition-metal-doped zinc sulfide phosphor, a common example of an ML material [39]. Upon mechanical stimulation of zinc sulfide phosphor, the doped transition metal ions are integrated into the crystal lattice of zinc sulfide, substituting for some zinc ions. Transition metal ions typically possess unpaired d-electron configurations. As the transition metal ions become excited, d-electrons transition from higher to lower energy levels, emitting photons in the process. The energy and wavelength of the emitted photons are determined by the energy level differences associated with the transition, resulting in varying emission wavelengths from the zinc sulfide material due to different transition metal ions. This emitted light propagates via total internal reflection along the optical fiber, with a demodulator at the terminus detecting the intensity and wavelength characteristics of light and generating a spectrum. Analysis of this spectrum can then indirectly infer the parameters of the physical quantity being measured.

### 2.3. Optic Fiber Fabry–Perot Sensor

The optical fiber Fabry–Perot sensor operates on the principle of Fabry–Perot interference. Current fabrication methods for Fabry–Perot sensors include the chemical etching method, which introduces bubbles into the fiber to create interference microcavities via a chemical reaction [40,41,42]; the laser processing method that employs femtosecond lasers to produce microcavities [43,44,45]; the direct welding method, which involves welding single-mode fibers with special fibers to form microcavities with gaps [46,47,48,49]; and the polymer-assisted filling method, which incorporates polymer materials into special fibers to create sealed air cavities during direct welding [50,51].

The working principle of the Fabry–Perot sensor, depicted in Figure 2c, involves using light propagation and reflection within the optical fiber to measure object parameters. A light source is connected to one end of the fiber, and reflections from the end faces of the cavity create an interference signal that varies with the length and refractive index of the cavity. Environmental changes can cause cavity deformation, resulting in a phase shift of the interference pattern. Consequently, Fabry–Perot sensors can measure a range of parameters, including humidity [52], temperature [53], curvature [54], acoustics [55], and strain [56].

A unique type of Fabry–Perot sensor, known as the diaphragm-type optical fiber sensor, features a fiber end that acts as the primary reflection point and a diaphragm that serves as the secondary reflection point. The diaphragm is constructed from materials like metal or polymers with desirable flexibility and deformability, which serve as sensing elements. This sensor responds to external stimuli to measure various physical quantities, including pressure, deformation, and temperature. The operating principle of the diaphragm-type optical fiber sensor is depicted in Figure 2d. The sensing principle involves the thin film making direct contact with the object under investigation by being attached to the end face of the optical fiber or encircling it. This vibration induces a change in the distance between the film and the end surface, thereby modulating the phase of the emitted light, which allows for the measurement of the physical quantity. This sensor responds to external stimuli to measure various physical quantities, including pressure, deformation, and temperature. During the measurement of physical quantities [57], the relationship between the deflection of the diaphragm h and the pressure P applied by a sound wave or pulse is given by

(3) $$h=\frac{3(1-v^{2})Pa^{4}}{16Et^{3}}.$$

Here, v is the Poisson ratio of diaphragm, E is Young’s modulus, P is the pressure induced by a pulse or sound signal, t is the thickness of the diaphragm, and a is the radius. Due to the pressure caused by the sound wave or pulse signal acting on the diaphragm, deformation occurs. This deformation modulates the phase of the light by altering the propagation path of the light at the end face of the optical fiber:

(4) $$\Delta \varphi =\frac{4\pi nh}{\lambda }$$

where λ represents the central wavelength of the incident light and n denotes the refractive index of the fiber core.

Fabry–Perot sensors leverage interferometric principles to offer significant advantages in accurately measuring physical quantities, including temperature, pressure, and refractive index. The diaphragm-type optical fiber sensor, characterized by its flexibility, facilitates real-time monitoring and recording of acoustic cardiovascular conditions [57], imaging [58], and pulse waves [59] in patients during rehabilitation treatment. The fiber optic Fabry–Perot sensor holds broad application prospects in wearable rehabilitation devices, offering personalized and precise rehabilitation services to patients.

### 2.4. Polymer Optical Fiber Sensor

Polymer optical fiber sensors are constructed from polymer materials for the purpose of optical signal transmission and sensing. In comparison to traditional silica fibers, polymer fibers offer greater flexibility, lower refractive indices, and heightened sensitivity, thus providing distinct advantages for their use in optical fiber sensing applications [60]. The fabrication of these sensors generally encompasses three primary steps:

- Polishing: Specialized equipment and techniques are employed to polish the polymer optical fiber, and 3D printing may be utilized [61] to create smooth and sensitive regions, ensuring a smooth fiber surface for effective interaction with external media. Within this process, parameters such as the polishing length, depth, and curvature radius can influence the sensitivity of the sensor.
- Cladding Treatment: an appropriate cladding treatment is applied to the polished region to maintain optimal optical characteristics and mechanical integrity.
- Fixed Encapsulation: the polished optical fiber is securely encapsulated to safeguard the polished region and establish a connection with the sensor system.

The operating principle of the polymer fiber sensor, as depicted in Figure 2e, is based on the principles of refraction and total internal reflection within the fiber. As light enters the polymer fiber and reaches the sensitive region, a portion is refracted into the external medium, while the remainder continues along the path of the fiber. Variations in the external refractive index of the medium, temperature, pressure, or other physical and chemical properties will result in changes to the intensities of both the refracted and transmitted light within the polished area. By measuring these variations in light intensity, the corresponding changes in the parameters of the object can be determined.

Presently, polymer optical fiber sensors are being utilized in the creation of curvature and joint motion sensors within the wearable rehabilitation treatment sector. These devices offer reliable medical data on physiological parameters, motion status, and muscle activity, which aids in the development and refinement of rehabilitation protocols [60].

### 2.5. Long-Period Fiber Grating Sensor

Long-Period Fiber Grating (LPFG) sensors are a type of optical sensor that utilizes optical fibers as the sensing medium. They achieve sensitive detection of external physical quantities—such as temperature, pressure, and bending—by introducing periodic refractive index variations within the optical fiber.

The working principle of LPFG sensors is based on the coupling effect between optical waveguide modes in the fiber. In an LPFG, periodic refractive index changes are introduced into the cladding of the optical fiber, forming a grating structure. As light propagates along the fiber and passes through the grating area, light of a specific wavelength resonates with the grating, leading to energy exchange between specific modes of the grating. This energy exchange results in an attenuation peak, known as the resonance peak, in the transmission spectrum for light of a particular wavelength. The resonant wavelength is related to the period of the grating, the effective refractive index of the fiber, and the refractive index of the surrounding environment. When environmental conditions change, such as temperature or pressure variations, the effective refractive index of the grating also changes, leading to a shift in the resonant wavelength. By monitoring the shift in the resonant wavelength, changes in external physical quantities can be detected.

The fabrication method for LPFG sensors typically involves the following steps [62,63]:

- Fiber Selection: choose an appropriate single-mode or specialty optical fiber as the base material.
- Grating Fabrication: use techniques such as ultraviolet light, CO_2_ lasers, or femtosecond lasers to inscribe periodic refractive index variations on the fiber, creating the LPFG structure.
- Chemical Etching: sometimes, chemical etching is required to adjust the parameters of the grating, such as by etching the outer cladding to enhance sensitivity to environmental conditions.
- Functional Coating: apply a specific material, like graphene oxide, onto the grating surface to provide chemical functionality, facilitating subsequent immobilization of biomolecules or other types of sensing applications.

LPFG sensors, due to their high sensitivity, compatibility, and integrability, have a wide range of applications in various fields such as biomedical science and rehabilitation therapy [64,65,66].

### 2.6. Distributed Optical Fiber Sensor

Distributed Optical Fiber Sensors (DOFSs) are a type of sensor technology that utilizes optical fibers as the sensing medium to continuously, and in real time, monitor physical quantities (such as temperature, strain, vibration, etc.) along the length of the optical fiber.

The working principle of DOFS is mainly based on the light scattering effects in the optical fiber, including Rayleigh scattering, Brillouin scattering, and Raman scattering. These scattering effects interact with the light waves propagating in the optical fiber, causing changes in the frequency, phase, intensity, etc., of the light waves. These changes are related to the changes in physical quantities along the fiber. By measuring these changes, the distribution of physical quantities along the fiber can be inferred [67,68].

Rayleigh scattering: elastic scattering caused by tiny density fluctuations in the optical fiber, with the scattered light frequency being the same as the incident light, is commonly used for distributed temperature and vibration monitoring.

Brillouin scattering: Inelastic scattering caused by the interaction of sound waves in the optical fiber, resulting in frequency changes, is used for measuring temperature and strain.

Raman scattering: inelastic scattering caused by molecular vibrations in the optical fiber material, producing Stokes and anti-Stokes light, is used for temperature monitoring.

The fabrication of Distributed Optical Fiber Sensors involves the selection of optical fibers, the preparation of the fibers, and the integration of the sensor system. The choice of optical fiber depends on the monitoring parameters required and the environmental conditions. For example, single-mode fibers are typically used for Rayleigh and Raman scattering sensors, while multi-mode or few-mode fibers may be used for Brillouin scattering sensors. The preparation of optical fibers includes drawing the fibers, coating, and possible special treatments such as doping or the creation of FBGs [69].

Distributed Optical Fiber Sensors are widely used in various fields such as healthcare and environmental monitoring, but they still face some challenges, including cost, spatial resolution, measurement range, and the capability for multi-parameter measurement [18,70].

### 2.7. Micro-/Nanofibers Sensor

Micro-/Nanofiber (MNF) sensors are a highly sensitive sensing technology that utilizes micro/nanoscale optical fibers as the sensing medium to achieve continuous, real-time monitoring of physical quantities (such as temperature, strain, pressure, humidity, etc.) along the length of the fiber. The working principle of MNF primarily relies on various effects produced by the interaction of light with matter within the optical fiber, including intensity, scattering, absorption, interference, and changes in waveguide modes. These effects are associated with changes in the physical quantities along the fiber, and by precisely measuring these changes, the distribution of physical quantities along the fiber can be monitored [71,72,73].

In terms of manufacturing methods, MNF sensors involve a variety of techniques [74,75]:

- Electrospinning technology: This process uses high voltage to eject a polymer solution into fine fibers, creating fibers at the micro/nano-scale.
- Flame heating method: used for preparing silicon dioxide MNFs, this method heats standard optical fibers with a flame, gradually stretching and reducing their diameter.
- Laser heating method: A CO_2_ laser beam serves as the heat source for preparing MNFs with excellent surface smoothness and uniform diameter within a micro furnace.
- Chemical synthesis and nano-lithography: these techniques are used for fabricating polymer MNFs and include methods such as chemical synthesis, electrospinning, and physical drawing.

MNFs have been widely applied in fields such as healthcare, environmental monitoring, and industry [76,77,78].

### 2.8. Performance Comparison of Wearable Optical Fiber Sensors

We can explore three main aspects: a comparison of advantages and disadvantages, materials and costs, and production and manufacturing processes.

- Comparison of Advantages and Disadvantages: Self-luminescent, stretchable optical fiber sensors are superior in applications requiring no external power, yet they come with higher production costs and design complexities. In contrast, FBGs and Fabry–Perot sensors excel in precision monitoring for structural health, temperature, and strain measurements, though they have high production costs and require precise manufacturing processes. Diaphragm-type and polymer fiber optic sensors, however, provide better cost-effectiveness owing to simplified production processes and cost-efficient materials. LPFGs and DOFs offer highly sensitive detection of external physical quantities, but their practical use may require complex signal processing and data analysis.
- Materials and Costs: Material selection is crucial; self-luminescent, stretchable optical fiber sensors and FBG sensors often necessitate specific high-performance materials, influencing production costs. Fabry–Perot sensors utilize precision materials, yet their varied designs can accommodate cost control, to some extent. Diaphragm-type and polymer fiber optic sensors typically employ cost-effective, easily processed materials like polydimethylsiloxane (PDMS) and polycarbonate (PC). Material selection for LPFGs and DOFs hinges on sensing requirements and the ability to adapt to environmental conditions. Overall, material selection and cost management are pivotal in determining the broad application of sensors.
- Production and Manufacturing Processes: The unique nature of self-luminescent, stretchable optical fiber sensors demands intricate mechanical design and meticulous manufacturing processes. Producing FBGs and Fabry–Perot sensors requires sophisticated laser writing and precise controls, somewhat restricting their large-scale manufacturing. Diaphragm-type and polymer fiber optic sensors can utilize industrial polishing, 3D printing, and injection molding, thus lowering production barriers and costs. LPFGs fabrication entails creating periodic refractive index changes, potentially using UV exposure, CO_2_ lasers, or chemical etching. DOF production hinges on optical fiber selection, preparation, and sensor system integration and may include special treatments like doping or FBG fabrication.

## 3. The Application of Penetrable Optical Fiber Sensors in Finger Parameter Detection

Hand diseases are prevalent health issues that can significantly impact one’s quality of life. Yet, accurate motion guidance and detection are especially critical during rehabilitation sessions. Proper exercise guidance can prevent sports injuries and expedite the rehabilitation process for patients with hand diseases. By monitoring patients’ exercises, medical personnel can promptly identify irregularities, providing timely adjustments and guidance to prevent unnecessary injuries resulting from improper exercise techniques during rehabilitation. Hand rehabilitation often necessitates the use of various assistive devices and technologies for movement monitoring and guidance. Nevertheless, advancements in science and technology have introduced optical fiber sensors as a novel solution for hand rehabilitation challenges. Optical fiber sensors can be adeptly integrated into hand rehabilitation for the monitoring and guidance of hand movements. For instance, embedding optical fiber sensors within rehabilitation aids allows for real-time monitoring of hand movements and targeted guidance based on data analysis.

### 3.1. Detection of Finger Movement

#### 3.1.1. Gesture Recognition

Gesture recognition technology is becoming increasingly vital in rehabilitation, aiding in the recovery of motor function for stroke and hand injury patients. Conventional rehabilitation typically depends on manual guidance and assessment from physiotherapists, a process that is time-consuming, labor-intensive, and challenges the feasibility of continuous monitoring and tailored training. The advent of wearable optical fiber sensors has revolutionized rehabilitation treatment. These sensors can accurately capture and record finger movements and joint angle changes, enabling real-time gesture recognition through computer-aided analysis. Furthermore, when integrated with virtual reality and game-based systems, wearable optical fiber sensors can offer a more diverse and engaging range of training modalities, enhancing rehabilitation efficiency.

In the realm of gesture recognition, wearable optical fiber sensors leverage their high sensitivity to accurately detect hand movements and joint changes, enabling precise identification of complex gestures. This capability for discerning nuanced movements not only underpins applications in virtual reality, gaming interaction, and assistive communication but also introduces innovative approaches to rehabilitation medicine and athletic training [79,80,81,82]. Bai et al. introduced a novel, stretchable lightguide for multimodal sensing (SLIMS) [31], consisting of a series of parallel elastomeric optical fibers featuring continuous or discrete ribbon patterns. SLIMS can discern and quantify the position, extent, and nature of mechanical deformations—such as stretching, bending, or pressing—utilizing the principles of frustrated total internal reflection and absorption. By integrating SLIMS sensors into a wireless glove, they enabled multi-point and multi-mode deformation decoupling, allowing for real-time tracking of a comprehensive range of finger joint movements and external pressures.

**Figure 3:**
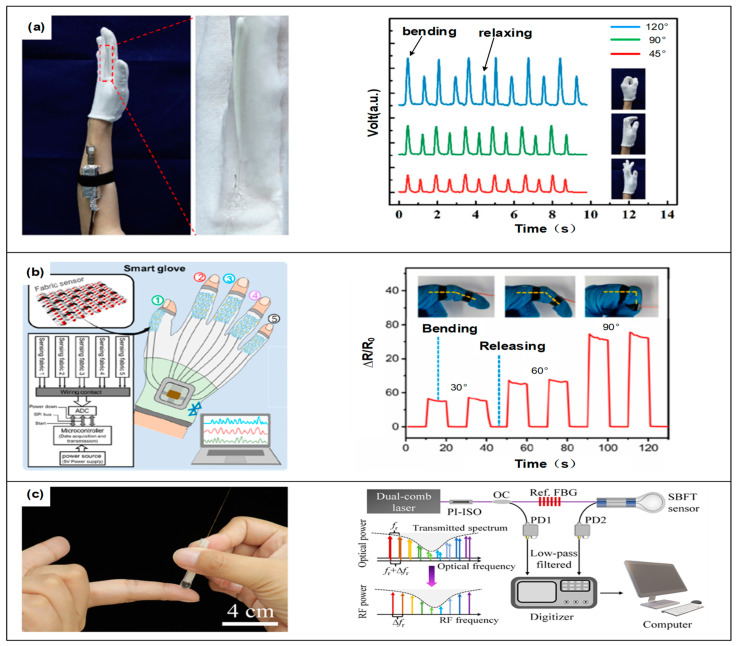
Application diagram of optical fiber sensor in detecting finger parameters. (**a**) Self-powered, stretchable mechanoluminescent optical fiber strain sensor, courtesy of Ref. [67]. (**b**) Structure diagram and detection diagram of stretchable optical strain sensor, courtesy of Ref. [74]. (**c**) Application and the schematic diagram of a soft, bio-inspired, fiber optic tactile sensor (SBFT), courtesy of Ref. [83].

Liang et al. introduced a self-powered, stretchable mechanoluminescent optical fiber strain sensor [83], as shown in Figure 3a. By integrating a mechanoluminescent (ML) phosphor with an elastomeric optical fiber, the sensor emits visible light upon external stretching or release, eliminating the need for an external light source or power supply. The sensor demonstrates a linear strain response of up to 50% and a high precision strain measurement capability, achieving an accuracy of 1%. Additionally, they showcased the application of the sensors in wearable gloves and implantable devices for detecting human motion. For instance, integrating the sensors into gloves allows for monitoring finger joint movements, while those implanted in tissue, such as that of pig, can track muscle movements. Yang et al. introduced a self-powered photoelectric cooperative fiber sensor (SOEFSs), an optical fiber electronic device capable of visualizing and digitizing mechanical stimulation without the need for an external power supply [84]. The sensor responds to mechanical stimulation through a combination of mechanoluminescence (ML) and the triboelectric effect. SOEFSs feature a unique sheath–core structure, with the sheath composed of an optically active composite material and the core made of electrically active material. Utilizing a thermoplastic ML material system and spinning process, an industrial-grade, continuous manufacturing and recycling process for SOEFSs has been established, realizing sustainable production.

The application of optical fiber sensors in gesture recognition demonstrates their unique advantages. The basic composition of a gesture recognition system typically encompasses three core components: an optical fiber sensor array, a signal acquisition and processing module, and a pattern recognition algorithm. Through the synergistic operation of these components, the system can accurately capture and recognize a wide array of gestures.

#### 3.1.2. Measurement of Finger Joint Angle

In daily human activities, from delicate operations to routine typing, precise control and measurement of finger joint angles are essential. Detecting finger joint angles is crucial for assessing an individual’s athletic capacity and health status and is also indispensable in fields such as rehabilitation medicine, physical training, ergonomics, and virtual reality. Sensors can accurately measure joint flexion by monitoring the propagation changes of light within optical fibers, thereby acquiring precise joint angle data [85,86,87,88]. A key advantage of this technology is its minimal susceptibility to environmental interference and non-irritating nature to the skin, allowing for prolonged wear and real-time monitoring. Li et al. introduced an optical neuron (MPON) utilizing a silicon-based microfiber probe for detecting and recognizing the motion of robotic fingers [89]. Silicon-based microfiber probes are fabricated by drawing biconical, silicon-based optical microfibers from standard single-mode fibers and embedding them within layers of PDMS. To safeguard the waveguide structure of the microfiber probe and protect against environmental contamination, a PDMS–Teflon–Microfiber–Teflon–PDMS composite structure was developed. With this composite structure, the MPON is capable of accurately measuring a wide range of bending angles with a rapid response.

Qu et al. introduced a high-sensitivity, stretchable optical strain sensor designed for monitoring human activity and providing medical care [90]. The structural and detection diagrams for this sensor are presented in Figure 3b. The sensor employed a simple, cost-effective design with unique mechanical and sensing properties, allowing for integration into clothing or direct attachment to the skin to monitor a range of human activities, from subtle physiological signals like wrist pulses to large joint movements and gestures. The sensor is capable of measuring large strains, up to 100%, with a low detection limit of 0.09%, a rapid response time of less than 12 ms, and high repeatability exceeding 6000 cycles.

The application of optical fiber sensors in detecting knuckle bending angles reveals their potential in biomedical engineering. The flexible optical fiber sensor, when placed close to the skin, can accurately detect minute changes in the knuckles. The signal processing unit converts the collected sensor data into an electrical signal and performs initial amplification and filtering. Data analysis software employs advanced algorithms to interpret the signals, enabling the precise calculation of knuckle bending angles.

### 3.2. Detection of Finger Physiological Parameters

Optical fiber sensors offer an effective means for the precise monitoring of physiological parameters, including finger movement, blood flow, and muscle activity. Furthermore, the wearability of optical fiber sensors allows for extended monitoring without disruption to patients’ daily routines, which is crucial for enhancing rehabilitation efficiency and patient experience.

#### 3.2.1. Blood Oxygen Saturation and Pulse Waveform

Oxygen saturation indicates the level of oxygenated hemoglobin in the blood, and the pulse waveform offers insights into the pumping function of the heart and the condition of the blood vessels. During rehabilitation treatment for conditions like heart disease, chronic obstructive pulmonary disease, and sleep apnea syndrome, monitoring blood oxygen saturation and pulse waveforms is crucial for guiding treatment and preventing potential complications [91]. For instance, continuous monitoring of blood oxygen saturation can assist physicians in evaluating the respiratory support requirements of the patient, and alterations in the pulse waveform may signal changes in cardiac load or enhancements in vascular elasticity. Optical fiber sensors are capable of continuously and accurately collecting physiological data without disrupting patients’ daily activities. For instance, Shweta Pant et al. [92] introduced a novel, non-invasive finger plethysmography technique utilizing a Fiber Bragg Grating sensor to capture arterial pulse waveforms. This approach differs from the traditional pulse oximetry method, which assesses not only oxygen saturation in the blood but also records the actual pulse pressure waveform experienced by the arterial wall, offering a more comprehensive assessment of cardiovascular health.

Concurrently, the FBG Plethysmographic Pulse Recorder (FBGPPR), a device they designed, can dynamically record volume changes in the fingers due to pulsatile blood flow in the ulnar artery. Data from the FBGPPR were compared against the radial artery pulse waveform (RAPPW). as recorded using the FBG-based pulse recorder (FBGPR). Furthermore, the study illustrates the use of the FBGPPR and an electronic stethoscope to assess pulse transmission times and analyzes the captured pulse waveforms, which can reveal subtle changes and significant features within them. The characteristics are closely associated with factors such as vascular aging, arterial stiffness, the atherosclerosis index, and the effects of vasoactive drugs.

The application of optical fiber sensor technology enhances both the quality and efficiency of monitoring and offers patients a more comfortable and convenient rehabilitation experience. As technology advances, the use of optical fiber sensors in rehabilitation is expected to expand, offering increasingly personalized and efficient treatment options to patients.

#### 3.2.2. Skin Temperature

In rehabilitation treatment, finger temperature serves as a physiological indicator that reflects the state of local blood circulation and tissue metabolic activity. Assessing normal finger temperature indices can facilitate wound healing, alleviate pain, prevent infections, and accelerate the rehabilitation process. For instance, during hand trauma or post-surgical rehabilitation, monitoring finger temperature can help to identify circulatory disorders and prevent complications in a timely manner. Utilizing optical fiber sensors for temperature monitoring enables continuous, real-time tracking of finger temperatures and offers scientific data to support rehabilitation treatments [93,94].

Liu et al. [95] introduced a ratiometric fluorescence optical fiber sensor that utilizes integrated silicate and tellurite glass for real-time human thermal monitoring. Within this sensor, Er^3+^/Yb^3+^ co-doped tellurite glass generates up-conversion fluorescence emissions associated with thermal coupling energy levels, enabling temperature measurements through near-infrared excitation. The molten tellurite glass was drawn into a silicate tube using a negative pressure technique, achieving tight adhesion between the dissimilar glass materials, which have notably different softening temperatures. The sensor can be placed in the mouth or on the skin of the hand to continuously monitor body temperature. Additionally, the sensor can be deployed in the oral exhalation and nasal breathing environment for real-time thermal activity monitoring, offering a novel and highly reliable approach for human health surveillance. The research indicates that the sensor boasts high sensitivity, rapid response, a compact design, robust packaging, low cost, and high reliability, making it potentially valuable for real-time life and health monitoring applications.

Fiber optic sensors have demonstrated considerable value in the detection of finger physiological parameters, particularly in the real-time tracking of critical indicators like blood oxygen saturation, pulse waveform, and body temperature. These sensors provide physicians with crucial health data through high-precision measurements, aiding in the assessment of treatment efficacy and the adjustment of rehabilitation strategies. Optical fiber sensor technology is progressing towards greater sensitivity, enhanced durability, and reduced costs.

### 3.3. Detection of Finger Touch

In the rehabilitation field, advancements in optical fiber sensor technology have introduced new possibilities for tactile detection. By quantifying the effects of temperature, pressure, and slip on the fingers, these sensors can offer comprehensive tactile feedback [96,97,98,99]. This multi-parameter monitoring assists healthcare professionals in gaining a deeper understanding of patients’ rehabilitation processes, thereby optimizing treatment plans and enhancing the personalization of care.

Shang and colleagues [100] introduced a soft, bio-inspired, fiber optic tactile sensor (SBFT) capable of sensing and differentiating between temperature and pressure akin to human skin, as shown in Figure 3c. The SBFT sensor is constructed by encapsulating a macroscopically curved Fiber Bragg Grating (FBG) within an elastomer droplet, generating two distinct types of optical resonances—FBG-related and whispering gallery modes (WGMs)—which propagate along the curvature. By leveraging the distinct thermo-optical and stress-optical properties of FBG and WGM resonances, the SBFT sensor can fully decouple pressure and temperature measurements, achieving an accuracy of 0.2 °C for temperature and 0.8 mN for pressure.

Li et al. introduced a skin-like optical fiber tactile sensor (SOFT), inspired by artificial intelligence, designed to emulate the tactile signal generation and processing functions of human skin [101]. It possesses multifunctional tactile interaction capabilities, including the detection of tactile amplitude, position, and tensile strain. The stretch unit of the SOFT features a unique “double X” configuration, enhancing the stretchability of the sensor, with tactile units aligned in a straight line for ease of array partitioning. The SOFT is composed of four FBG sensors embedded within a three-layer structure resembling skin, creating a flexible tactile sensing array capable of over 20% stretchability. Utilizing a two-stage cascade neural network, the SOFT can concurrently discern the position and magnitude of applied forces. Within the 0–3.5 N force range, the SOFT achieves a contact position recognition accuracy of 92.41%, with an error margin of less than 4.2%.

Optical fiber sensor technology mimics human tactile responses by precisely monitoring critical tactile indicators, like pressure, slip, and temperature, and sensitively detecting and quantifying external stimuli. This aids medical professionals in assessing treatment efficacy and refining treatment plans.

## 4. Challenges and Future Research Directions of Wearable Optical Fiber Sensors

We summarized the applications, types of optical fiber sensors, and performance of the various fiber optic sensors listed in Table 1. Currently, the majority of wearable optical fiber sensors utilized for finger parameter recognition are not yet extended for providing feedback in human health assessments or for on-demand robotic assistance. As noted in Ref. [102], the integration of wearable optical fiber sensing systems into exoskeleton assistive devices remains in the conceptual phase. Looking ahead, a key research direction for optical fiber sensors is to develop a compact, fully integrated, and cost-effective monitoring system suitable for clinical settings. However, current optical fiber sensing systems lack the necessary integration, making them less competitive than electronic sensors with regard to widespread clinical application. Numerous challenges persist, including sensor structural design, optimal measurement site selection, signal acquisition, multi-parameter measurement capabilities, and production costs, among others.

When designing wearable optical fiber sensors for finger parameter recognition, several key considerations should be addressed:

- They must exhibit high sensitivity and flexibility.
- The structure of the sensor must accurately detect changes at the measurement point, conform to the joints and muscles, accommodate the full range of human finger motion, and account for the effects of relative displacement and friction at the sensor-to-skin interface on measurement accuracy.
- Employing flexible encapsulation or manufacturing techniques, along with a thoughtful arrangement of optical fiber sensors, can fulfill the durability requirements necessary for wearable applications.
- It is essential to analyze the mechanical properties of flexible materials, ensure the stability and repeatability of their performance for long-term monitoring, and address the hysteresis inherent in flexible materials, which can affect measurement accuracy [103,104]. Hysteresis compensation techniques should be integrated to enhance the precision of the measured parameters.

At present, while fiber optic sensing technology, particularly FBG and Fabry–Perot sensors, excels in functional performance, it encounters challenges in practical application, such as high costs, large detection equipment size, and overall transportation difficulties, which limit their extensive use in clinical and rehabilitation settings. Nevertheless, as science and technology advance, we anticipate significant future transformations of these sensing systems. First, during deployment, beyond the typical issues like temperature, vibration, and dust, numerous environmental elements can impact the performance and precision of fiber optic sensors. These include humidity, electromagnetic interference, chemical contaminants, pressure variations, UV radiation, and air pollutants. These can irreversibly impair signal acquisition, transmission, measurement accuracy, sensitivity, and the integrity of the fiber material. Measures to combat these factors are mainly reflected in the protection with special coatings and encapsulation materials, the implementation of fiber optic isolation techniques to electrically insulate the optical sensor from subsequent electronic components, and the application of ultraviolet-resistant materials and coatings. Thus, employing innovative structures, materials, and design approaches is essential for sensor miniaturization, stable signal transfer, and improved detection capabilities. These innovations broaden the applicability of fiber optic sensors, facilitating precise measurements even in challenging conditions [105,106,107]. Second, combining multidimensional modeling with machine learning provides a novel approach to parameter decoupling. Machine learning has demonstrated significant potential in gait phase recognition and prediction. Integrating artificial intelligence and big data analysis allows for in-depth sensor data analysis, facilitating accurate diagnoses and personalized treatment plans [108,109,110]. Third, through the optimization and refinement of query algorithms, sensor performance can be enhanced while simultaneously reducing complexity and cost [111,112,113,114]. A notable advantage of wavelength-modulated optical fiber sensors is their capability to measure multiple parameters using a single optical fiber. This capability is particularly crucial for scenarios requiring the simultaneous monitoring of multiple biomechanical parameters. Additionally, phase- and intensity-modulated optical fiber sensors are capable of multi-parameter measurement, offering enhanced possibilities for temporal and spatial monitoring. The advancement of distributed optical fiber sensing technology, particularly two-dimensional sensing, offers novel solutions for high-resolution measurements of position and pressure. Compared to traditional quasi-distributed measurements, two-dimensional sensing technology exhibits superior spatial resolution and accuracy, providing significant advantages for monitoring human biomechanical parameters. Concurrently, advances in wireless technology are expected to improve the convenience and real-time capabilities of fiber optic sensor data collection [110,115]. These technologies enable comprehensive patient analysis and assessment, promoting the development of more precise treatment plans.

Currently, GaAs-based, phosphor, MEMS, and Distributed Optical Fiber sensors have been commercially available for years and hold substantial market value. In the future, the design and manufacturing of wearable fiber optic sensors must balance performance with cost-effectiveness. Advancements and innovations in technology will likely make future fiber optic sensors more efficient and cost-effective, expanding their use in clinical and rehabilitation fields [116,117,118,119,120].

## 5. Conclusions

To summarize, this short review provided a comprehensive overview of the applications and advancements of wearable optical fiber sensors within rehabilitation, aligning with the requirements of wearable sensor technology in the rehabilitation field and leveraging the benefits of optical fiber sensors. Optical fiber sensors hold significant promise in rehabilitation treatment due to their high sensitivity, interference resistance, and portability. This review has described the operating principles and manufacturing techniques of various optical fiber sensors, including self-luminous stretchable sensors and FBG sensors, and discussed their applications in monitoring finger movements, physiological parameters, and tactile sensations. Additionally, the review has highlighted the challenges of current technology, including higher sensitivity and lower costs, and outlined potential future research directions. It is anticipated that wearable optical fiber sensors will become commercialized and find widespread application in rehabilitation therapy.

## Figures and Tables

**Table 1:** Measurement of human finger information based on optical fiber sensing.

Reference	Application	Type of Optical Fiber Sensors	Performance
[80]	Gesture recognition	SMF Sensor	Capable of recognizing up to 12 basic gestures with an accuracy rate as high as 94%.
[85]	Finger joint angle	POF Sensor	The sensitivity can reach 0.070 dB/°.
[87]	Pulse waveform	SCF Sensor	At pressures below 200 Pa, the sensitivity is 2.2 kPa^−1^; within the pressure range of 200–600 Pa, the sensitivity is 0.91 kPa^−1^.
[91]	Oxygen saturation and Heart rate	POF Sensor	Capable of rapidly responding to external pressure changes, with a response time of 5 milliseconds.
[93]	Temperature monitoring	MNF Sensor	Demonstrated a temperature sensitivity as high as −30 nm/°C. Capable of achieving a resolution of 0.0012 °C.
[96]	Tactile and temperature monitoring	FBG Sensor	Tactile sensitivity of 7.287 nm/MPa. Temperature sensitivity of 13 pm/°C.
[82]	Touch sensing and body temperature	FBG Sensor	A pressure sensitivity of 0.03 nm/kPa, a bending angle sensitivity of 0.19 nm/°, and a temperature sensitivity of 0.04 nm/°C.

## Data Availability

No new data were created or analyzed in this study. Data sharing is not applicable to this article.
